# Plasma Adrenomedullin level in Egyptian children and Adolescents with type 1 diabetes mellitus: relationship to microvascular complications

**DOI:** 10.1186/1758-5996-2-12

**Published:** 2010-02-10

**Authors:** Safinaz A El-Habashy, Randa M Matter, Eman S El-Hadidi, Hala R Afifi

**Affiliations:** 1 Department of Pediatrics, Faculty of Medicine, Ain Shams University, Cairo 11566, Egypt; 2 Department of Clinical Pathology, Faculty of Medicine, Ain Shams University, Cairo 11566, Egypt

## Abstract

**Background:**

Adrenomedullin (AM) is known to be elevated in different clinical situations including diabetes mellitus (DM), but its potential role in the pathogenesis of vascular complications in diabetic children and adolescents is to be clarified. Hence, the study aimed at assessment of plasma adrenomedullin levels in children and adolescents with type 1 DM and correlation of these levels with metabolic control and diabetic microvascular complications (MVC).

**Methods:**

The study was performed in the Diabetes Specialized Clinic, Children's Hospital of Ain Shams University in Cairo, Egypt. It included 55 diabetic children and adolescents (mean age 13.93 ± 3.15 years) who were subdivided into 40 with no MVC and 15 with MVC. Thirty healthy subjects, age-and sex- matched were included as control group (mean age 12.83 ± 2.82 years). Patients and controls were assessed for glycosylated hemoglobin (HbA1c) and plasma adrenomedullin assay using ELISA technique.

**Results:**

Mean plasma AM levels were significantly increased in patients with and without MVC compared to control group, (110.6 pg/mL, 60.25 pg/mL and 39.2 pg/mL respectively) (P < 0.01) with higher levels in those with MVC (P < 0.05). Plasma AM levels were positively correlated with both duration of diabetes (ρ = 0.703, P < 0.001) and glycemic control (HbA1c) (ρ = 0.453, P < 0.001).

**Conclusion:**

Higher plasma AM levels in diabetics particularly in those with MVC & its correlation with diabetes duration and metabolic control may reflect the role of AM in diabetic vasculopathy in the pediatric age group.

## Background

Adrenomedullin (AM), a ubiquitous regulatory peptide with different actions, is expressed in many tissues throughout the body including the adrenal medulla, endothelial [1] and vascular smooth muscle cells [2], myocardium and central nervous system [3,4]. Adrenomedullin (AM) is widely synthesized and secreted from most of the cells in the body [5]. It controls proliferation, differentiation and migration of cells [6]. Adrenomedullin (AM) is able to act as an autocrine, paracrine, or endocrine mediator in a number of biologically significant functions [7]. It plays a critical role in several diseases such as cancer, diabetes, cardiovascular and renal disorders [8,9]. It has vasodilator and blood pressure lowering properties and plays important role in maintaining electrolyte and fluid homeostasis [10]. Endogenous AM may protect from organ damage by inhibiting oxidative stress production [11] and raised AM levels correlated with increased oxidative stress [12]. Moreover, evidence that AM possesses a clear cut proangiogenic effect under both physiological and pathophysiological conditions has accumulated [13-15]. Adrenomedullin is involved in insulin regulatory system [16-18] and is elevated in plasma from patients with pancreatic dysfunctions such as type 1 or type 2 diabetes and insulinoma [18]. Adrenomedullin might play a role in the pathogenesis of diabetic vasculopathy in type 1[19] and type 2 diabetes [20]. However, to the best of our knowledge, there is no published data about AM level in type 1 diabetic children and adolescents. Hence, this study aimed at assessment of plasma adrenomedullin levels in type 1 diabetic children and adolescents and correlating levels with metabolic control and diabetic microangiopathy.

## Materials and methods

### Subjects

This case control study included 55 consecutive type 1 diabetic children and adolescents recruited from Diabetology Clinic, Children's Hospital, Ain Shams University, Cairo, Egypt during the period from May 2004 to May 2006. Those with liver disease, renal failure or congestive heart failure were excluded [19]. According to the presence or absence of MVC, patients were classified into: Group 1: 40 diabetic patients without MVC. Group 2 comprised 15 diabetic patients with MVC (retinopathy, neuropathy and/or nephropathy). Thirty apparently healthy age and sex matched children and adolescents were included as a control group. Informed consent was obtained from patients' parents or their legal guardians after study approval by the Local Ethical Committee, Ain Shams University (FWA00006444).

### Methods

Patients were subjected to careful history taking laying stress on onset, duration, frequency of diabetic ketoacidosis (DKA) or hyperglycemic attacks, thorough clinical examination with special emphasis on signs of diabetic complications. Fundus examination was performed by an ophthalmologist after maximum papillary dilatation using indirect ophthalmoscope to identify diabetic retinopathic changes [21].

### Laboratory investigations

Assessment of glycemic control by calculating the mean glycosylated hemoglobin (HbA1c) over the last year was performed using high performance liquid chromatography (HPLC) technique [22]. Patients were considered under optimal glycemic control when their HbA1c was < 7.5% [23]. Microalbuminuria was assayed using SERA-PAK immuno-microalbumin Kit (Bayer Corporation, Benedict Ave, Tarry town, NY, USA). Persistent microalbuminuria was defined when two of three samples showed urinary albumin excretion rate of 30-300 μg/mg creatinine [24]. Two ml of venous blood were collected on EDTA tube, centrifuged for 15 minutes and plasma samples were stored at -70°C till assay. Plasma adrenomedullin level was assessed by ELISA technique using adrenomedullin (human) (EIA-3418) kit, DRG international Inc., USA).

### Statistical Analysis

Analysis of data was performed by using SPSS (version 15). Comparison between 2 groups of patients was made using Student's t-test for parametric measures and Wilcoxon signed-rank test (Z value) for non parametric measures. Spearman's rank correlation coefficient was used to correlate between two quantitative variables. P value < 0.05 was considered the cut-off value for significance.

## Results

Diabetic patients (n = 55) and controls were comparable as regards age, gender and BMI. Age, duration of diabetes and mean HbA1c were significantly higher in patients with MVC (n = 15) compared to patients without MVC (n = 40) (P < 0.01, P < 0.01, P < 0.05 respectively) and to controls (Table 1). Compared to healthy subjects, each patients' group displayed significantly increased AM levels (P < 0.05), with higher values in diabetics with MVC than those without (P < 0.05) (Table 1, Table 2). Significant positive correlation between AM levels and both duration of diabetes (ρ = 0.703, P < 0.001) and HbA1c (ρ = 0.453, P < 0.001) was observed among diabetic patients (n = 55) with a non significant correlation with age (ρ = 0.09, P = 0.51).

**Table 1:** Comparison between diabetic patients with and without MVC and controls as regards age, disease duration and laboratory findings.

Variable Tests	Group 1 Patients with MVC (N = 15)	Group 2 Patients without MVC(N = 40)	Controls (N = 30)	
Male/Female	7/8	26/14	17/13	

	Mean ± SD	Mean ± SD	Mean ± SD	P

Age (yrs)	17.13 ± 1.5	12.73 ± 2.7	12.83 ± 2.4	P1 < 0.001, P2 < 0.001, P3 > 0.05*

BMI(Kg/m2)	21.39 ± 3.25	23.27 ± 3.8	22.11 ± 3.23	P > 0.05*

Systolic BP percentile	70.48 ± 21.7	66.4 ± 26.4	67.5 ± 23.23	P > 0.05*

Diastolic BP percentile	68.84 ± 13.6	64.7 ± 19.8	66 ± 18.3	P > 0.05*

Duration of diabetes (yrs)	9.6 ± 3.3	3.8 ± 3.8	-	P < 0.001*

HbA1c (%)	9.36 ± 1.8	8.09 ± 2.02	4.2 ± 0.4	P < 0.05*

AM (pg/mL)	110.6 ± 94.24	60.25 ± 31.76	39.2 ± 12.43	Z1-2.01, Z2-2.7, Z3-3.37 P1 = 0.04, P2 < 0.01, P3 = 0.001#

SD, standard deviation; BMI, body mass index;*Student t test; # Wilcoxon signed-rank test (Z value), Z1, P1 between groups 1&2; Z2, P2 between groups 1 & controls; Z3, P3 between group 2 & controls.

**Table 2:** Median plasma adrenomedullin levels in patients with and without diabetic microangiopathy and control group

Group	N	Median AM(pg/ml)	Confidence interval*
Nephropathy	15	75	58.41-162.79

Nephropathy + retinopathy	5	200	23.02-348.98

Nephropathy + neuropathy	3	70	

Without MVC	40	65	50.22 - 70.28

Controls	30	40	34.64 - 43.76

*95^th ^confidence interval

## Discussion

In the present study, diabetic patients showed highly significant increase in plasma AM levels compared to controls. These results are in agreement with Hayashi et al [25] who reported significant increase in plasma AM in hyperglycemic patients compared with normal volunteers. However, Kinoshita et al. [26] found that when patients with nephropathy were excluded, plasma levels of AM were not significantly different in old diabetic patients and healthy individuals.

Our patients with MVC displayed higher AM levels compared to those without. Similar results were reported in other studies [19,20,27]. Yet, they reported no significant difference in patients with nephropathy, neuropathy or retinopathy (P > 0.05). In the current study, highest AM levels were observed in diabetic patients with retinopathy and nephropathy. Higher plasma AM levels in our diabetic patients with microalbuminuria compared to normoalbuminuric patients differs from Garcia-Unzueta et al [19] who reported higher levels of AM and cAMP in patients with renal insufficiency but normal in microalbuminuric patients. Furthermore, adult type 1 diabetic patients with renal insufficiency had higher levels of plasma AM than diabetics with other complications, and plasma AM increase was proportionate to kidney function deterioration [19]. Adrenomedullin could exert a wide range of vascular actions (mostly protective). These include endothelium-dependent and -independent vasodilatation, antioxidative stress, stimulation of endothelial nitric oxide production, antiproliferation of vascular smooth muscle cell, and adventitial fibroblast[28]. Taken together, the elevation of plasma adrenomedullin level in type 1 diabetes(especially in the presence of nephropathy) could participate in the mechanism against progression of vascular damage [28].

Highest individual plasma AM values recorded in our diabetics with retinopathy are similar to previous studies [19,29]. Adrenomedullin may also play a role in the neovascularization process that occurs after retinal ischemia. Increased AM levels in vitreous humor of patients with proliferative vitreoretinopathy [30,31] and diabetic retinopathy [32] suggested the involvement of AM as a possible associated factor in the course of vascular and proliferative retinal diseases.

Increased AM levels with longer duration of diabetes is consistent with Garcia-Unzueta et al. [19] who reported similar relationship suggesting that the elevation of AM levels is a late phenomenon due to endothelial dysfunction. Also, there was a significant correlation between AM levels and HbA1c with higher HbA1c levels among diabetics with MVC. Similarly, Caliumi et al. [33] reported that increased circulating AM correlates with poor glucose metabolic control in type 2 diabetics. The elevated plasma AM level originates from vascular AM expression induced by hyperglycemia through protein kinase C-dependent pathway [34].

Our patients with MVC displaying higher AM levels were older than those without. However, no significant correlation was observed between AM and age. Similarly, Hayashi et al [25] reported no change in AM levels with age. Older age, longer duration of diabetes and puberty are known risk factors for MVC [35]. It is still uncertain whether increased release of AM in diabetes is a compensatory mechanism or a coincident event. The precise role of AM in the pathogenesis of diabetic complications is still to be elucidated [36].

## Conclusions

The increase in plasma adrenomedullin level in type 1 diabetic children and adolescents being correlated with disease duration and metabolic control; the two most independent risk factors for the occurrence of MVC; may declare its role in the pathogenesis of diabetic microangiopathy since childhood.

## Abbreviations

AM: adrenomedullin; DM: diabetes mellitus; MVC: microvascular complications; HbA1c: glycosylated hemoglobin; DKA: diabetic ketoacidosis; ELISA: enzyme linked immune sorbent assay; cAMP: Cyclic adenosine monophosphate; ACE: angiotensin converting enzyme.

## Competing interests

The authors declare that they have no competing interests.

## Authors' contributions

SAE supervised the work and reviewed the manuscript. RMM designed the study, analyzed the data and drafted the manuscript. ISE carried out the laboratory studies. HRA collected the data. All authors approved the manuscript.
